# Chromosomal gains and losses in primary colorectal carcinomas detected by CGH and their associations with tumour DNA ploidy, genotypes and phenotypes

**DOI:** 10.1038/sj.bjc.6690388

**Published:** 1999-05-01

**Authors:** P M De Angelis, O P F Clausen, A Schjølberg, T Stokke

**Affiliations:** Institute of Pathology, The Norwegian National Hospital, 0027 Oslo, Norway; Department of Biophysics, Institute for Cancer Research, The Norwegian Radium Hospital, Oslo, Norway

**Keywords:** colorectal tumours, direct CGH, gains, losses, oncogenes, tumour suppressor genes

## Abstract

Comparative genomic hybridization (CGH) is used to detect amplified and/or deleted chromosomal regions in tumours by mapping their locations on normal metaphase chromosomes. Forty-five sporadic colorectal carcinomas were screened for chromosomal aberrations using direct CGH. The median number of chromosomal aberrations per tumour was 7.0 (range 0–19). Gains of 20q (67%) and losses of 18q (49%) were the most frequent aberrations. Other recurrent gains of 5p, 6p, 7, 8q, 13q, 17q, 19, X and losses of 1p, 3p, 4, 5q, 6q, 8p, 9p, 10, 15q, 17p were found in > 10% of colorectal tumours. High-level gains (ratio > 1.5) were seen only on 8q, 13q, 20 and X, and only in DNA aneuploid tumours. DNA aneuploid tumours had significantly more chromosomal aberrations (median number per tumour of 9.0) compared to diploid tumours (median of 1.0) (*P* < 0.0001). The median numbers of aberrations seen in DNA hyperdiploid and highly aneuploid tumours were not significantly different (8.5 and 11.0 respectively; *P* = 0.58). Four tumours had no detectable chromosomal aberrations and these were DNA diploid. A higher percentage of tumours from male patients showed Xq gain and 18q loss compared to tumours from female patients (*P* = 0.05 and 0.01 respectively). High tumour S phase fractions were associated with gain of 20q13 (*P* = 0.03), and low tumour apoptotic indices were associated with loss of 4q (*P* = 0.05). Tumours with *TP53* mutations had more aberrations (median of 9.0 per tumour) compared to those without (median of 2.0) (*P* = 0.002), and gain of 8q23–24 and loss of 18qcen-21 were significantly associated with *TP53* mutations (*P* = 0.04 and 0.02 respectively). Dukes' C/D stage tumours tended to have a higher number of aberrations per tumour (median of 10.0) compared to Dukes' B tumours (median of 3.0) (*P* = 0.06). The low number of aberrations observed in DNA diploid tumours compared to aneuploid tumours suggests that genomic instability and possible growth advantages in diploid tumours do not result from acquisition of gross chromosomal aberrations but rather from selection for other types of mutations. Our study is consistent with the idea that these two groups of tumours evolve along separate genetic pathways and that gross genomic instability is associated with *TP53* gene aberrations. © 1999 Cancer Research Campaign


*British Journal of Cancer* (1999) **80**(3/4), 526–535
© 1999 Cancer Research Campaign
Article no. bjoc.1998.0388

### Chromosomal gains and losses in primary colorectal carcinomas detected by CGH and their associations with tumour DNA ploidy, genotypes and phenotypes

**PM De Angelis[1], OPF Clausen[1], A Schjølberg[1] and T Stokke[2]**

[1]Institute of Pathology, The Norwegian National Hospital, 0027 Oslo, Norway; [2]Department of Biophysics, Institute for Cancer Research, The Norwegian Radium Hospital, Oslo, Norway

**Summary** Comparative genomic hybridization (CGH) is used to detect amplified and/or deleted chromosomal regions in tumours by mapping their locations on normal metaphase chromosomes. Forty-five sporadic colorectal carcinomas were screened for chromosomal aberrations using direct CGH. The median number of chromosomal aberrations per tumour was 7.0 (range 0–19). Gains of 20q (67%) and losses of 18q (49%) were the most frequent aberrations. Other recurrent gains of 5p, 6p, 7, 8q, 13q, 17q, 19, X and losses of 1p, 3p, 4, 5q, 6q, 8p, 9p, 10, 15q, 17p were found in > 10% of colorectal tumours. High-level gains (ratio > 1.5) were seen only on 8q, 13q, 20 and X, and only in DNA aneuploid tumours. DNA aneuploid tumours had significantly more chromosomal aberrations (median number per tumour of 9.0) compared to diploid tumours (median of 1.0) ($P < 0.0001$). The median numbers of aberrations seen in DNA hyperdiploid and highly aneuploid tumours were not significantly different (8.5 and 11.0 respectively; $P = 0.58$). Four tumours had no detectable chromosomal aberrations and these were DNA diploid. A higher percentage of tumours from male patients showed Xq gain and 18q loss compared to tumours from female patients ($P = 0.05$ and 0.01 respectively). High tumour S phase fractions were associated with gain of 20q13 ($P = 0.03$), and low tumour apoptotic indices were associated with loss of 4q ($P = 0.05$). Tumours with *TP53* mutations had more aberrations (median of 9.0 per tumour) compared to those without (median of 2.0) ($P = 0.002$), and gain of 8q23–24 and loss of 18qcen-21 were significantly associated with *TP53* mutations ($P = 0.04$ and 0.02 respectively). Dukes' C/D stage tumours tended to have a higher number of aberrations per tumour (median of 10.0) compared to Dukes' B tumours (median of 3.0) ($P = 0.06$). The low number of aberrations observed in DNA diploid tumours compared to aneuploid tumours suggests that genomic instability and possible growth advantages in diploid tumours do not result from acquisition of gross chromosomal aberrations but rather from selection for other types of mutations. Our study is consistent with the idea that these two groups of tumours evolve along separate genetic pathways and that gross genomic instability is associated with *TP53* gene aberrations.

**Keywords:** colorectal tumours; direct CGH; gains; losses; oncogenes; tumour suppressor genes

The molecular genetic model for colorectal carcinogenesis emphasizes the accumulation of, and sequence of, genetic aberrations in the development of sporadic colorectal carcinomas from adenomas (Fearon and Vogelstein, 1990). The aberrations identified thus far include deletions and/or point mutations of several important tumour suppressor genes such as *APC*, *DCC* and *TP53* (Bodmer et al, 1987; Fearon et al, 1987; Solomon et al, 1987; Vogelstein et al, 1988; Muleris et al, 1990; Powell et al, 1992; Meling et al, 1993; Miyaki et al, 1994) and mutations of oncogenes such as *K-ras* (Bos, 1989; Giaretti et al, 1996). Although genetic instability resulting from mutations may occur in all colorectal carcinomas, it seems unlikely that all have the same genetic evolutionary pattern, since there exist distinct differences in the histopathological features, distribution, clinical behavior and molecular characteristics of precursor lesions and invasive tumours (reviewed in Ilyas and Tomlinson, 1996; Houlston and Tomlinson, 1997).

Colorectal carcinomas can be grouped into two ploidy classes by flow cytometry measurements of tumour DNA content. DNA aneuploid tumours, including hyperdiploid, highly aneuploid and tetraploid tumours, have a stem line with abnormal DNA content, and DNA diploid tumours have normal cellular DNA content (Hiddemann et al, 1984). The calculated tumour DNA index is the ratio of $G_0/G_1$ peak channels of the tumour cells to normal (reference) cells and can be assumed to reflect the tumour karyotype, since there is good agreement between DNA index and chromosome number as determined by karyotypic analysis in human tumours and tumour cell lines (Tribukait et al, 1986; Bigner et al, 1987). Approximately 60% of all colorectal adenocarcinomas are DNA aneuploid, which often results in a poorer prognosis for the patient than if they are DNA diploid (Rognum et al, 1991; Bauer et al, 1993).

Flow cytometric measurements of tumour DNA content cannot elucidate the specific numerical and structural aberrations that occur in tumours. A technique that allows simultaneous screening of the entire tumour genome for chromosomal gains and losses was developed in 1992 (Kallioniemi et al, 1992) and called comparative genomic hybridization (CGH). CGH allows detection of amplified and/or deleted chromosomal regions in tumours (corresponding to putative oncogenes and/or tumour suppressor genes respectively) by mapping their locations on normal metaphase chromosomes, and has been used to screen for amplifications and deletions in several types of human neoplastic disease

*Received 22 July 1998*
*Revised 23 October 1998*
*Accepted 4 November 1998*

*Correspondence to:* PM De Angelis

**Table 1** Chromosomal gains and losses detected by CGH in 45 colorectal tumours

| Tumour | Gender | Dukes' stage | DI | Gains | Losses |
|---|---|---|---|---|---|
| 90–8 | M | C | 1.00 | 20q | |
| 90–10 | F | B | 1.00 | | |
| 92–4 | M | B | 1.00 | | |
| 93–3 | F | B | 1.00 | 8pter-q13 | 4pter-q26, 6q16.2–23 |
| 93–11 | F | B | 1.00 | | |
| 94–3 | M | B | 1.00 | X | 9p13–21 |
| 94–5 | F | B | 1.00 | 7, 8q21.2-ter, 13q, 17q21.3-ter, 20 | 3p, 4p, 5qcen-21, 8p21.1-ter, 15qcen-22.2, 17p13, 18q22-ter |
| 94–14 | M | B | 1.00 | 20q | |
| 94–17 | M | B | 1.00 | 20 | 8, 18 |
| 94–19 | F | D | 1.00 | | X |
| 94–22 | M | C | 1.00 | X | |
| 94–25 | F | B | 1.00 | | |
| 94–26 | F | B | 1.00 | 19p | 4qcen-24 |
| 94–27 | M | B | 1.00 | 7p, 13q, 19, 20 | 1pcen-32.2, 4q22-ter, 18qcen-21 |
| C896 | F | C | 1.10 | 8q, 17q, 20q | 1p13.3–31, 1q24–32.1, 2p12–23, 2q21.2–33, 4, 5, 6qcen-25.1, 8p21.3-ter, 18, Xp21.3-ter |
| 92–9 | F | B | 1.15 | Xq | 9p |
| 92–29 | F | B | 1.16 | 8q13–21, 8q24, 16p, 19, 20q | 1p21–31, 4pcen-14, 5q23–31 |
| 94–23 | F | B | 1.20 | 20q | 5q14–21, X |
| C1340 | M | D | 1.20 | 7p, **8q,** 13q, 17q, **20q, Xq** | 1q24–31, 4, 5q14-ter, 6qcen-22, 8p, 9p21-ter, 10qcen-25, 17p13-ter, 18 |
| C1402 | M | C | 1.20 | 7, 8p21.3-ter, 16q21-ter, 19, 20q | 1p31.1, 2q24.2–31, 4q, 10p 12-ter, 18 |
| 92–6 | F | D | 1.31 | 7, 9q, **13q,** 20 | 1pcen-22, 4, 5q31.3-ter, 9p23-ter |
| 92–2 | M | D | 1.37 | 7, **8q,** 11p14-ter, 13q, 17q21.2-ter, **20, Xq** | 1, 4, 5q14-ter, 8p, 9q, 10, 12q, 14q, 17p12-ter, 18q, 21q, 22q |
| 93–6 | F | C | 1.42 | 13qcen-12, 16p, 20q, X | 4, 6q15–23, 9p, 10q, 11q13.5-ter, 18q, 20p12-ter |
| 93–8 | M | C | 1.49 | 7, 13q, 20q | 1p21–32, 2, 4, 6qcen-23, 9, 11q14.2-ter, 14q13-ter, 15q24-ter, 18, 20p13, X |
| 90–17 | M | B | 1.50 | 7, **8q,** 9q, 13qcen-14.2, 20q13, Xpter-q21.2 | 4, 5q, 6qcen-25, 8p21.1-ter, 13q21-ter, 15q, 16q, 18q |
| 94–12 | M | C | 1.51 | 1q23-ter, 3, 4p, 5p, 7, 8q, 11q14-ter, 13q, 19q, 20, 21q, 22q, X | 15q, 17p, 18qcen-12.3 |
| 93–9 | M | B | 1.54 | 7, 8q, 11p15.1-ter, **20** | 1pcen-34, 2p13-ter, 3p23-ter, 4q31.2-ter, 5q13–31.2, 8p21.1-ter, 10p, 12p12-ter, 15q, 18q |
| 92–26 | F | C | 1.57 | 5p, 8q24, 13q32-ter | 6q21–23, 14q, 15qcen-22, 17pcen-12, 18q, 21, 22q12.3-ter |
| 94–15 | M | C | 1.58 | 5p, 9, 20q13 | 15q, 17p, 18q, 21q |
| 94–13 | M | B | 1.59 | 13q32-ter, X | |
| 92–8 | M | D | 1.60 | 2q22-ter, 7, 12q, 13q, 16q22-ter, 17q21.3-ter, 20q, X | 1p22-ter, 4, 8p, 11q21-ter, 15q15-ter, 17p12-ter, 18 |
| 94–28 | M | B | 1.63 | 6p23-ter, 7p, 13q, 16p, **20**, X | 1p, 4, 5qcen-32, 15q, 17p, 18q |
| 93–2 | M | C | 1.66 | 1q, 6pcen-22, 16p, 20, Xqcen-21, Xq25–26 | 1p21–22.3, 4q24–31.1, 15qcen-22, 18 |
| 92–30 | M | B | 1.67 | 8q23-ter, 13q, 20, X | |
| 94–18 | M | C | 1.69 | 5p, 6p21.2-ter, 7p, 8q21.1-ter, 11q13–22.1, 16q22-ter | 3p, 4, 5q11.2–32.3, 8pter-21.2, 18, 19pter-q13.1 |
| 92–1 | M | C | 1.70 | 7p15-ter, 8q24, 11qcen-14.3, 12, 13q, 17q22-ter, 20, Xq | 3p, 6q, 8p22-ter, 18q |
| 94–24 | M | D | 1.70 | X | 9p23-ter |
| 94–21 | M | D | 1.74 | 20q13 | |
| 95–2 | M | B | 1.75 | 6p21.1-ter, 6q25-ter, 8q23-ter, 9q33-ter, 13q, 20q | 10, 17pter-q21, 18 |
| 94–9 | M | B | 1.78 | 13q, 19q, 20 | 8p, 18qcen-21 |
| 93–5 | M | B | 1.83 | 4p14-ter, 5p14-ter, 8q, 20q | |
| 95–1 | M | B | 1.91 | 8q, **13q,** 20q | 8p21.1-ter, 15q, 17p, 18, 20p12-ter |
| 94–8 | M | C | 1.94 | 19p13.2 | |
| 94–10 | F | D | 1.97 | 13q32-ter, 20q | |
| 94–33 | M | | 2.22,1.61 | 5p13.2–14, 6p21.2-ter, 7, **8q,** 13q, 17q, **20**, X, **Xp** | 1p34.1-q41, 3, 4p, 5qcen-33, 6q14–25.2, 9q, 10, 12. 15q, 18q, 21q |

High-level gains (ratios > 1.5) are typed in bold print. M = male; F = female.

(Kallioniemi et al, 1992; Cher et al, 1994; Kallioniemi et al, 1995; Arnold et al, 1996; Korn et al, 1996; Heselmeyer et al, 1997; Tirkkonen et al, 1998). Recent CGH investigations of colorectal cancer (Ried et al, 1996; Nakao et al, 1998) have used indirect fluorescence methods to analyse relatively small series of primary colorectal carcinomas (16 and nine tumours respectively).

A characterization of the possible cytogenetic differences between DNA aneuploid and diploid tumours could result in a better understanding of differences in their respective biologies/behaviours. We screened a series of 45 sporadic unfixed colorectal carcinomas for chromosomal aberrations using a direct CGH method, which uses tumour and reference DNA probes that are directly conjugated to specific fluorochromes. Direct CGH improves the accuracy and reliability of CGH analysis compared to earlier, indirect methods (Karhu et al, 1997). DNA aneuploid and diploid colorectal tumours were analysed for possible differences

© Cancer Research Campaign 1999

in the type and frequency of recurrent chromosomal aberrations. We investigated possible associations of chromosomal aberrations with specific genotypes and phenotypes measured for this tumour set, and with several clinicopathological parameters.

### MATERIALS AND METHODS

### Tumour material

Forty-five colorectal carcinomas that had been surgically-removed and immediately frozen at –80°C were used for CGH analysis. The tumours were previously graded according to Dukes' stage (23 were Duke's B, 13 were Dukes' C and eight were Dukes' D; one tumour was not classified). Thirty tumours were obtained from male patients and 15 tumours from female patients. Gender and Dukes's stage information for this tumour group are presented in Table 1.

Most of the tumours used in this study were previously analysed for DNA content (De Angelis et al, 1993, 1995) using flow cytometry and the method of Vindeløv et al (1983) (DNA indices for each tumour are listed in Table 1). CGH analysis was done using DNA extracted directly from tumour samples re-analysed for DNA content on a FACSVantage laser flow cytometer (BDIS, San Jose, CA, USA) (21 cases) or using DNA extracted from fresh tumour tissue upon receipt of the tumours (24 cases).

### Criteria for inclusion of tumours in CGH study

A recent study (Kallioniemi et al, 1994) has demonstrated the importance of having at least 50% tumour cells in samples analysed by CGH in order to ensure optimal detection of amplifications and deletions. In our study, DNA aneuploid tumour samples for CGH analysis generally contained more than 60% tumour cells (median 68%) as determined by DNA flow cytometry. This value was not possible to determine for DNA diploid tumours by flow cytometry. However, since estimates of percentages of non-tumour epithelial cells and infiltrating leucocytes from histological sections of DNA aneuploid and diploid tumours were consistent with the percentages quantitated for the diploid component in aneuploid tumours by flow cytometry, it was reasonable to assume that the majority of diploid tumours also contained more than 60% tumour cells.

### Comparative genomic hybridization

#### *DNA extraction*

The preparation of genomic DNA from colorectal tumour samples was done using a standard protocol for DNA isolation. Slices of tumour tissue were cut up into small bits in 2 ml proteinase-K (PK) digestion buffer (100 mM sodium chloride, 10 mM Tris-HCl pH 8.0, 25 mM EDTA pH 8.0, 0.5% sodium dodecyl sulphate (SDS), to which PK was added when ready to use; 50 µl of a 20 mg $ml^{-1}$ PK stock solution was added to 10 ml buffer). Alternatively, tumour cell suspensions stained for DNA flow cytometry were spun down to remove the propidium iodine (PI) staining solution, the pellets vortexed and resuspended in 2 ml PK digestion buffer. In both cases, samples were allowed to incubate at 50°C overnight with shaking. When nearly all of the cellular protein was degraded, the digest was deproteinized by successive extractions with phenol:chloroform:isoamyl alcohol 49.5:49.5:1 (Fluka, Buchs, Switzerland). The DNA was recovered by ethanol precipitation, dried and resuspended in TE buffer (10 mM Tris-HCl, 1 mM EDTA pH 8.0). Tumour DNA concentrations were determined by measuring the fluorescence of Hoechst 33258-stained samples in a fluorometer.

#### *Nick-translation and hybridization*

CGH was done using directly fluorochrome-conjugated DNAs, as described previously (Kallioniemi et al, 1994) with minor modifications. Briefly, 1 µg genomic DNA was nick-translated with 1 nmol each of dATP, dCTP, dGTP (Gibco BRL, Gaithersburg, MD, USA), and either Texas red-5-dUTP (DuPont NEN, Boston, MA, USA) for normal reference DNA or -fluoroscein isothiocyanate (FITC)-12-dUTP (DuPont NEN, Boston, MA, USA) for tumour DNA, at 15°C for 45–90 min with 9 units of DNA polymerase I (Gibco BRL, Gaithersburg, MD, USA and Promega, Madison, WI, USA) and 0.03 units DNase I (Gibco BRL). The reaction was stopped by heating at 70°C for 10 min. Probe fragment sizes were generally distributed in the range of 800–3000 basepairs, if not then the nick-translation was repeated with an adjusted reaction time.

The metaphase preparations for CGH hybridization were prepared according to routine procedures from PHA-stimulated, methotrexate-synchronized, peripheral blood lymphocytes. The latter were dropped onto slides in a room with 60–65% relative humidity and stored at –20°C in 100% ethanol or at 0°C in a nitrogen-flushed dessicator. Before hybridization, the slides were denatured for 3 min at 74°C in 70% formamide, 2 × saline–sodium citrate (SSC) (pH 7.0), dehydrated in a sequence of 70%, 85% and 100% ethanol, incubated in a PK solution (0.1 µg $ml^{-1}$ in 20 mM Tris-HCl per 2 mM calcium chloride, pH 7.5) for 7.5 min at room temperature, dehydrated in the same alcohol series, air-dried and placed in a 37°C warm room. The hybridization mixture was prepared by mixing 200–400 ng FITC-labelled tumour DNA, 200–400 ng Texas red-labelled normal DNA, 20 µg Cot-1 DNA (Gibco BRL), 1/10 vol, 3 M sodium acetate and 2 vol, 100% ethanol. Tumours from males were always hybridized with male reference DNA, and tumours from females with female reference DNA. The probe mixture was precipitated by centrifugation at 14 000 rpm for 30 min at room temperature, the supernatant decanted, and the pellets air-dried. DNA was then dissolved in 10 µl hybridization buffer (50% formamide, 10% dextran sulfate, 2 × SSC, pH 7.0), denatured at 70°C for 5 min and then placed in a 37°C warm room. The hybridization mixture was applied to the slide spot, the area covered by a coverslip and sealed with rubber cement. The hybridized spreads were incubated at 37°C in a humidified chamber for 2–3 days. After hybridization, the slides were subjected to three 10-min washes in 50% formamide per 2 × SSC (pH 7.0) at 45°C, followed by two 10-min washes in 2 × SSC at 45°C, one 10-min wash in 2 × SSC per 0.1% Triton X-100 at room temperature and, finally, one wash in distilled water. They were then dehydrated in 70%, 85%, 100% ethanol, air-dried and mounted using an anti-fade solution, Vectashield (Vector Laboratories, Burlingame, CA, USA), containing 0.2 µM DAPI.

#### *Microscopy and data analysis*

DAPI fluorescence and probe signals were observed sequentially with a Zeiss Axioplan fluorescence microscope equipped with a triple-pass emission filter (blue, green and red), a corresponding beam splitter and separate excitation filters (UV, 470–490 nm, 578 nm). All filters ('Pinkel 1' filter set) were obtained from

 © Cancer Research Campaign 1999

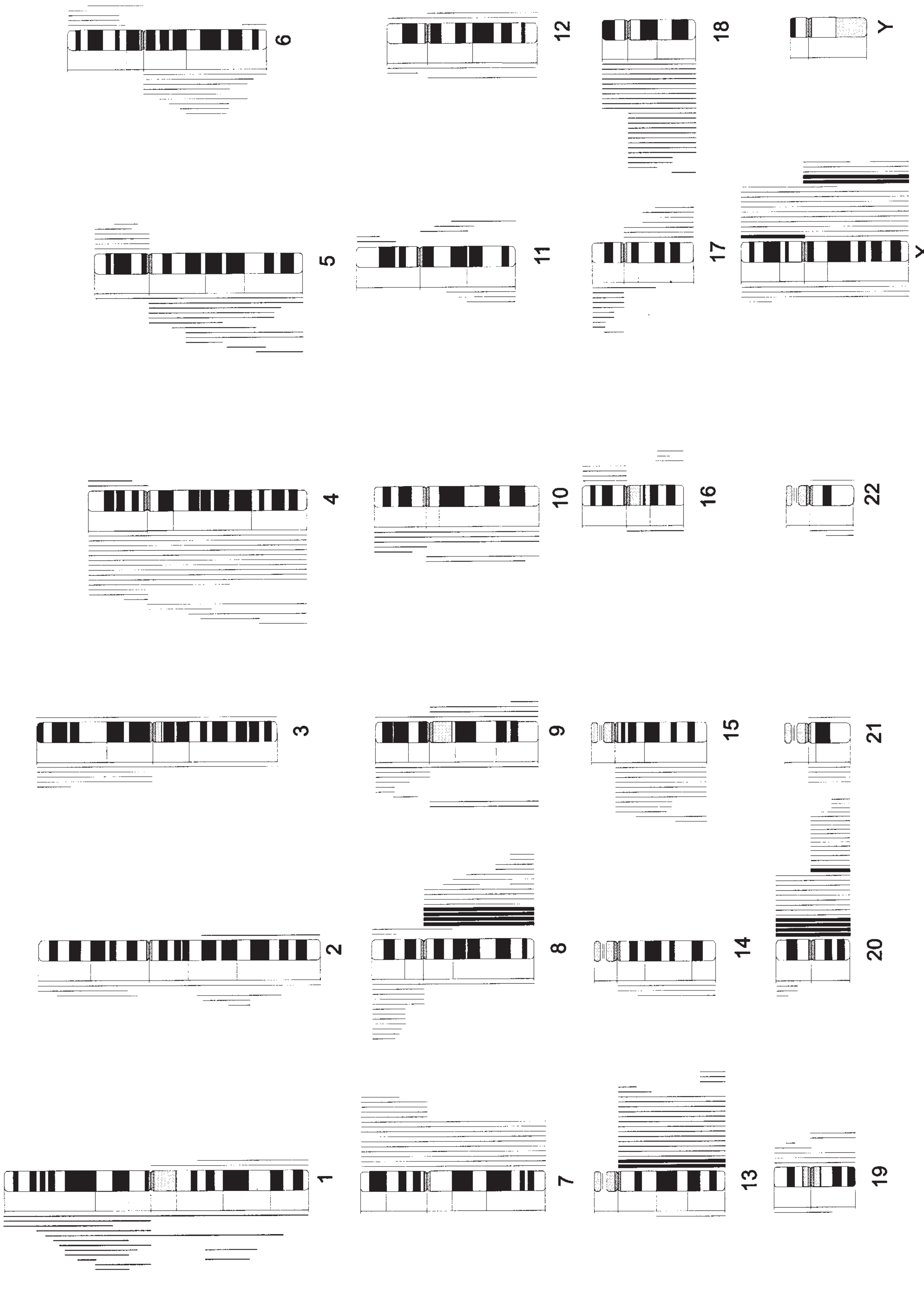

**Figure 1** Summary of chromosomal gains and losses in 45 colorectal carcinomas by direct CGH

© Cancer Research Campaign 1999

**Table 2** Associations of chromosomal aberrations with gender, Dukes' stage, tumour S phase fraction (SPF), apoptotic index (AI), *TP53* and K-*ras* genotypes

| Chromosome altered (No. detected) | Chromosomal arm altered (No. detected) | Minimal region of involvement (No. detected) | Association with gender (*P*-value) | Association with Dukes' stage (*P*-value) | Association with SPF (*P*-value) | Association with AI (*P*-value) | Association with *TP53* genotype (*P*-value) | Association with *K-ras* genotype (*P*-value) |
|---|---|---|---|---|---|---|---|---|
| 1 (16) | –1p (12) | 1p21–31.1 (12) | 0.72 | 0.30 | 0.76 | 1.00 | 0.48 | 0.46 |
| 4 (21) | –4q (16) | | 1.00 | 0.21 | 0.26 | **0.05** | 0.10 | 0.31 |
| 5 (18) | +5p (6) | | 0.65 | 0.18 | 0.38 | 0.66 | 0.40 | 0.16 |
| | –5q (12) | 5q14–32 (12) | 0.72 | 1.00 | 0.46 | 0.62 | 0.72 | 1.00 |
| 6 (14) | –6q (9) | 6q15–23 (9) | 0.70 | 0.13 | 0.25 | 1.00 | 0.12 | 0.11 |
| 7 (15) | +7p (15) | | 0.09 | 0.20 | 0.18 | 1.00 | 0.18 | 0.74 |
| 8 (30) | –8p (12) | 8p21.1-ter (12) | 0.28 | 1.00 | 0.34 | 0.62 | 0.06 | 0.50 |
| | +8q (16) | 8q23–24 (15) | 0.52 | 1.00 | 0.81 | 1.00 | **0.04** | 0.10 |
| 13 (21) | +13q (20) | 13q32-ter (18) | 0.33 | 0.53 | 0.65 | 0.69 | 0.10 | 1.00 |
| 15 (12) | –15q (12) | 15qcen-22 (11) | 0.29 | 1.00 | 0.09 | 0.68 | 0.45 | 0.70 |
| 17 (17) | –17p (10) | 17p12–13 (10) | 0.46 | 0.48 | 0.38 | 1.00 | 0.12 | 1.00 |
| | +17q (7) | 17q21-ter (6) | 1.00 | 0.09 | 0.31 | NE | 0.40 | 0.43 |
| 18 (23) | –18q (23) | 18qcen-21 (22) | **0.01** | 0.13 | 0.77 | 0.71 | **0.02** | 0.20 |
| 20 (33) | +20q (30) | 20q13 (30) | 0.09 | 0.54 | **0.03** | 0.44 | 0.08 | 0.74 |
| X (20) | +Xq (16) | | **0.05** | 0.52 | 0.39 | **0.03** | 1.00 | 1.00 |

Significant and marginally-significant associations observed were with male gender, Dukes' C/D, high SPF, low AI, *TP53* and K-ras mutations. One exception: gain of Xq was associated with high AI. NE = not evaluable.

Chroma (Brattleboro, UK). Images from 7–9 metaphases were captured and digitized in a cooled 16-bit black/white CCD camera (Astromed, Cambridge, UK).

Segmentation and calculation of ratio profiles were performed with CGH software (kindly provided by Damir Sudar), running under the 'Scilimage' image analysis program (TNO, Delft, The Netherlands) with Resource for Molecular Cytogenetic extensions (from Damir Sudar and Joe Gray, UCSF). This program segments metaphase chromosomes on the basis of the sum of the DAPI and Texas red images, subtracts background locally for each chromosome in the FITC and Texas red images, and calculates the intensity along each chromosome by integrating perpendicularly to the median axis. The total FITC and Texas red intensities for all chromosomes are used to normalize the intensities before calculation of the ratio between FITC and Texas red as a function of fractional length.

Normal reference DNA was also FITC-labelled and hybridized to normal reference DNA which had been TR-labelled in order to check that the green to red fluorescence intensity profiles for each chromosome were close to 1.0.

### *Criteria for hybridizations and for scoring tumour chromosomal aberrations*

Hybridization quality was assessed microscopically, and was generally considered to be acceptable if there was uniform strong hybridization over all metaphase spreads and if each spread generated consistent fluorescent intensity profiles. Hybridizations that resulted in low FITC or Texas red chromosomal fluorescence (signal to background ratio < 2), 'grainy' chromosome appearance, or poor to no blocking of the labelled probes to the centromere regions and the p-arms of acrocentric chromosomes, were repeated. Approximately 50% of the tumours were hybridized 2–3 times to obtain acceptable results.

The average and standard deviation of several (> 3) profiles of each chromosome were calculated, and more profiles were added until the averaged profile and standard deviation did not change after the addition of a new one. When using these criteria, ratios less than 0.85 and greater than 1.15 were never observed in normal versus normal hybridizations. Amplifications and deletions were therefore scored if the ratio was above 1.15 and below 0.85 respectively. These cut-off values are generally used for CGH analysis (Kallioniemi et al, 1994; Tirkkonen et al, 1998). Additionally, it was a requirement that the mean ratio plus one standard deviation did not exceed 1.0 for the deletions, and that the mean ratio minus one standard deviation was not below 1.0 for the amplifications. These last precautions ensured that inconsistent rises or declines in the ratio of single profiles (e.g. at the telomeres) were not scored as aberrations. Chromosome Y hybridization was generally weak, and possible aberrations on this chromosome were not scored. Apparent aberrations on the p-arms of acrocentric chromosomes and in centromere regions were not scored. It is important to note that, although the ratios may fluctuate in these regions, the normalization, and thereby the ratios in other 'unique' regions of the genome, are not much affected because the integrated intensities, rather than the integrated ratio, are used for normalization.

### Determination of tumour genotypes and phenotypes

*TP53* genotype/phenotypes were determined previously for this tumour set (De Angelis et al, 1993, 1995, 1998) using the techniques of constant denaturant gel electrophoresis (CDGE), DNA sequencing and immunoblotting. Forty tumours were also analysed for mutations in codons 12 and 13 of the *K-ras* gene using enriched polymerase chain reaction (PCR) techniques, restriction fragment length polymorphism analysis and direct sequencing as described previously (Andersen et al, 1997). DNA indices (De Angelis et al, 1993, 1995, 1997), S phase fractions (De Angelis et al, 1997) and apoptotic indices (AI; De Angelis et al, 1998), were determined previously for many of the tumours in this series.

### Statistical analyses

*T*-tests or Mann–Whitney two-tailed tests were used to check for significant differences between two data groups for a specific

 © Cancer Research Campaign 1999

parameter. Fisher's exact two-tailed 2 × 2 contingency test was used to check for associations between any two parameters, and Pearson or Spearman correlation analysis was used to check for the degree of covariation between two variables. All statistical testing was performed using Prism software (GraphPad Software, San Diego, CA, USA). *P*-values ≤0.05 were considered to be significant. In some instances with Fisher's exact test, the program reported marginally-significant associations (*P*-values ranging from 0.06 to 0.15).

### RESULTS

### Overview of genetic aberrations in colorectal carcinomas detected by CGH

The CGH results for 45 colorectal carcinomas are summarized in Figure 1 and Table 1. Chromosomal gains and losses are reported as recurrent aberrations if they were seen in at least five or more cases (> 10%) of 45 analysed. Four tumours had no detectable chromosomal aberrations. The median number of aberrations per tumour was 7.0 (range 0–19); the numbers of aberrations per tumour were distributed bimodally (Figure 2), with a natural cut-off at 6.0. The median number of gains per tumour was 3.0, as was the median number of losses. The number of gains per tumour correlated with the number of losses ($r = 0.58$, $P < 0.0001$). Gains of 20q (in 67% of tumours) and 13q (45%), and losses of 18q (49%) and 4q (36%) were the most frequent aberrations. Gains of 5p (13%), 6p (11%), 7p (33%), 8q (33%), 17q (16%), 19q (11%) and Xq (36%), and losses of 1p (27%), 3p (11%), 5q (27%), 6q (20%), 8p (27%), 9p (16%), 10q (11%), 15q (27%) and 17p (22%), were other recurrent aberrations. High-level gains (green to red ratio profiles >1.5) were seen only on chromosomes/chromosome arms 8q, 13q, 20 and X. The minimal regions of involvement (defined by a minimum of three tumours) for recurrent aberrations occurring in > 13% of the tumours are described in Table 2.

Thirty-one of 45 tumours were DNA aneuploid (DI > 1.0), and 14 tumours were DNA diploid (DI = 1.00). One aneuploid tumour had two stemlines, one with a DI of 1.61 and one with a DI of 2.22. DNA aneuploid tumours clearly had more chromosomal aberrations than diploid tumours, with a median number of aberrations per tumour of 9.0 (range 1–19) compared to 1.0 (range 0–12) in DNA diploid tumours ($P < 0.0001$) (Figure 3). DNA aneuploid and diploid tumours had similar types of chromosomal aberrations. The four tumours with no detectable chromosomal aberrations were all DNA diploid. Six aneuploid tumours were DNA hyperdiploid (1.00 < DI < 1.30) and 25 were highly DNA aneuploid (DI ≥ 1.30); these two groups did not have significantly different median numbers of aberrations per tumour (8.5 and 11.0 respectively; $P = 0.58$). Four aneuploid tumours were near-tetraploid/tetraploid (1.80 < DI ≤ 2.20), and these had a median number of 3.0 aberrations per tumour (range 1–8).

### Tumour genotypes and phenotypes

*TP53* mutations were detected in 27 of 42 colorectal tumours analysed for mutations, and 30 of 45 tumours were found to express p53 by immunoblotting. *K-ras* mutations were detected in 18 of 40 tumours analysed for mutations in codons 12 and 13 of the gene.

The distribution of S phase fractions for the tumour group was Gaussian and ranged from 5.5% to 23.7%, with a mean (±s.d.) of 14.0% ±4.6. The distribution of apoptotic indices for the tumour group was bimodal, and ranged from 0.0% to 5.4%, with a natural cut-off at 1.0%. Tumours with < 1.0% apoptotic cells (18 of 30 analysed) were designated as having a low AI, and tumours with ≥ 1.0% apoptotic cells (12 of 30) as those with a high AI for the purposes of statistical analyses.

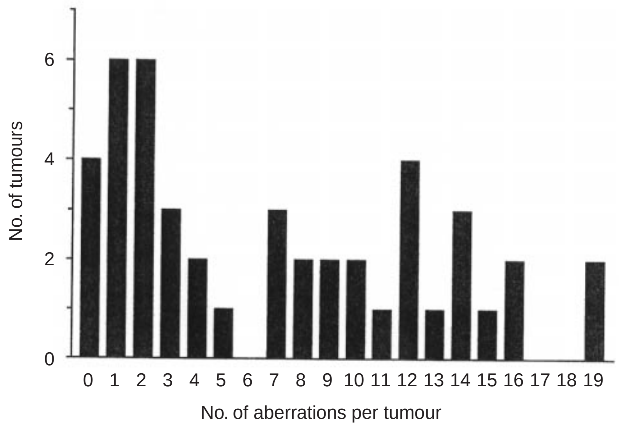

**Figure 2** Frequency distribution of the number of CGH aberrations per tumour for 45 colorectal carcinomas

### Associations of recurrent chromosomal aberrations with clinicopathological parameters, tumour genotypes and phenotypes

Table 2 summarizes the associations of the minimal regions of involvement with patient gender, Dukes' stage, tumour S phase fraction, tumour apoptotic index, *TP53* and *K-ras* genotypes.

Colorectal tumours from male patients had a median number of aberrations per tumour of 7.5 (range 0–19), whereas tumours from female patients had a median number of aberrations per tumour of 3.0 (range 0–13) ($P = 0.099$). Losses of 18q were detected in significantly more tumours from males (63%) compared to females (20%) ($P = 0.01$). Xq gains were also significantly associated with patient gender; 47% of tumours with Xq gain were derived from male patients compared to 13% from female patients ($P = 0.05$). Losses of/on chromosome X were detected in four tumours, three of which were from females; the tumour from a male with loss of X had a DNA index of 1.49.

Dukes' C/D stage tumours (metastasizing) tended to have a higher median number of aberrations per tumour compared to Dukes' B stage tumours (non-metastasizing), 10.0 (range 1–19) versus 3.0 (range 0–14) ($P = 0.065$) respectively. The proportion of detected aberrations which were designated as recurrent was 71% in Dukes' C/D tumours, compared to 88% in Dukes' B tumours (median values; $P = 0.437$). There were no significant associations of any recurrent chromosomal aberrations with Dukes' stage; however, Dukes' C/D compared to Dukes' B tumours tended to have more losses of 6q (29% to 9%) and 18q (62% to 35%) and more gains of 5p (19% to 4%) and 17q (24% to 4%) respectively.

Tumour S phase fractions were signficantly higher in colorectal tumours with gains of chromosome arm 20q13 (mean of 15.2% ±4.5) compared to tumours without 20q13 gain (mean of 12.0% ±4.0) ($P = 0.03$). Correspondingly, tumour S phase fractions tended to

© Cancer Research Campaign 1999

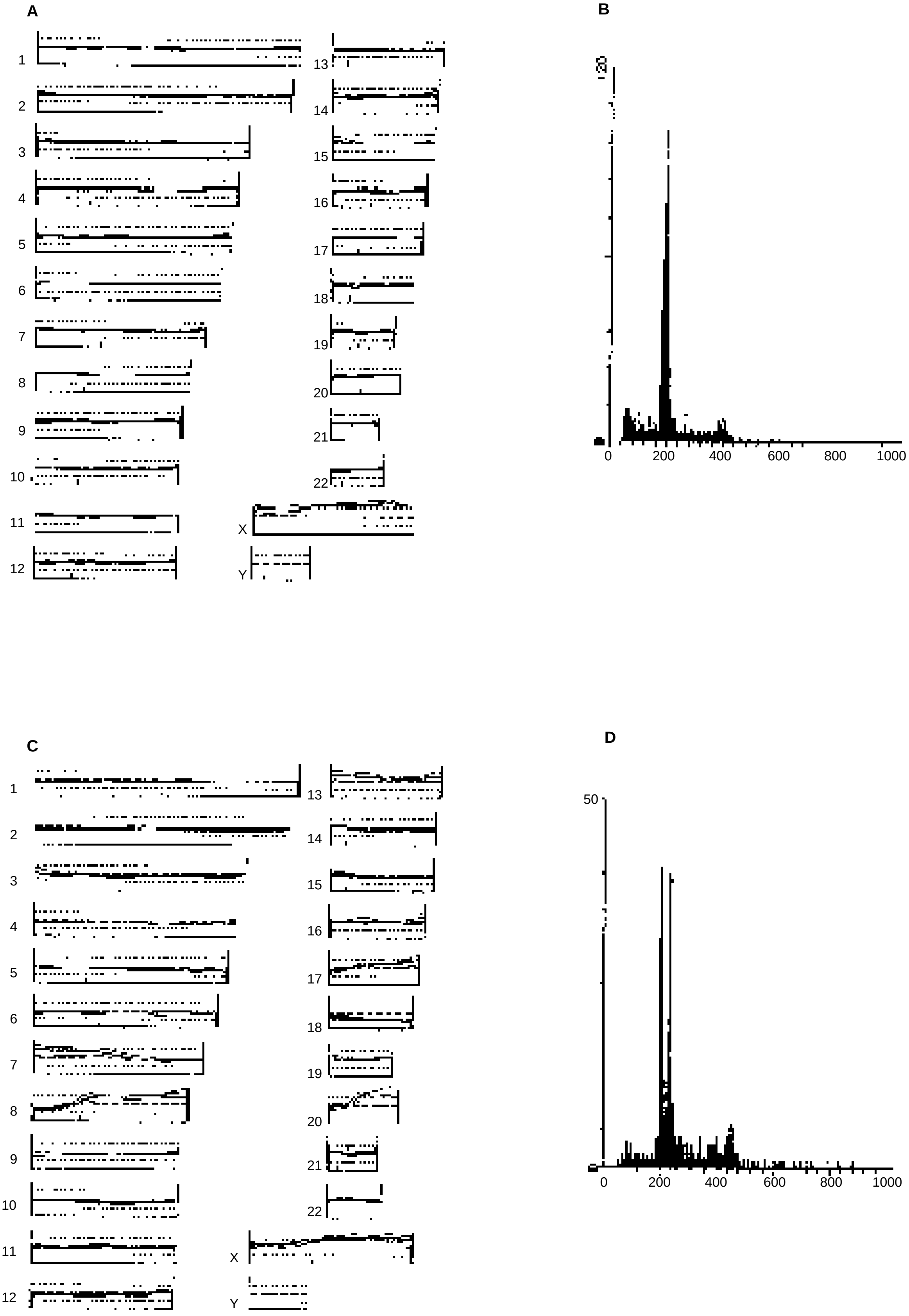

**Figure 3** CGH profiles for DNA diploid tumour 94–22 (**A**) and corresponding DNA histogram, DI = 1.00 (**B**); CGH profiles for DNA aneuploid tumour C1340 (**C**) and corresponding DNA histogram, DI = 1.20 (**D**). The *X*-axis of the DNA histograms is red-fluorescence (area) corresponding to tumour DNA content

 *© Cancer Research Campaign 1999*

be higher in tumours with 15qcen-22 loss than in tumours without this loss (*P* = 0.09).

Low tumour AI were significantly associated with loss of chromosome 4q, since 89% of tumours with 4q loss had low AI compared to 48% of tumours without 4q loss (*P* = 0.05). High AI were associated with Xq gain, since 75% of tumours with Xq gain had high AI compared to 27% of tumours without (*P* = 0.03).

The median number of aberrations per tumour in tumours without *TP53* mutations was 2.0 (range 0–19), compared to a median of 9.0 (range 0–19) in tumours with *TP53* mutations (*P* = 0.002). However, the median number of aberrations per tumour was not significantly different for p53-negative (3.0) compared to p53-positive (7.5) tumours (*P* = 0.295). Gains of 8q23–24 were significantly associated with *TP53* mutations, since 48% of tumours with *TP53* mutations had these gains compared to only 13% of tumours with wild-type *TP53* status (*P* = 0.04). Similarly, 67% of tumours with *TP53* mutations had losses of 18qcen-21 compared to 27% of tumours without mutations (*P* = 0.02). There were no significant associations of any chromosomal aberration with *TP53* phenotype.

The median numbers of chromosomal aberrations per tumour were not significantly different between tumours with and without *K-ras* mutations, 7.5 and 3.0 respectively (*P* = 0.253). There were no significant associations demonstrated between any chromosomal aberration and *K-ras* genotype.

### DISCUSSION

Our CGH results show that recurrent chromosomal aberrations in colorectal tumours are manifested as whole or partial gains of chromosomes/chromosome arms 5p, 6p, 7, 8q, 13q, 17q, 19q, 20q and Xq, and whole or partial losses of chromosomes/chromosome arms 1p, 3p, 4, 5q, 6q, 8p, 9p, 10, 15q and 18. These results are for the most part in agreement with those of Ried et al (1996; indirect CGH), except that their study did not report any gains on chromosome 19 or any losses on 6q, 10, or 15q. Additionally, the frequency of individual chromosomal losses in their study generally tended to be lower than in ours. These discrepancies may be due to the differences in detection sensitivity between the direct and indirect CGH methods and to the differences in cut-off values used when scoring chromosomal aberrations, or to the fact that they used formalin-fixed archival material. Our results are also consistent with the results of a karyotypic characterization of colorectal tumours by Bardi et al (1995), who reported the same gains and losses observed in the present study, but at lower frequencies for several individual chromosomal aberrations.

Gains of 20q13 and 13q32-tel and losses of 18qcen-21 and 4q were the most frequent aberrations seen in colorectal tumours. Genes that map to these locations include an unknown oncogene at 20q13 (Tanner et al, 1994), the *Smad2* tumour suppressor gene at 18q21 (Eppert et al, 1996) and the *Smad4*/*DPC4* and *DCC* tumour suppressor genes at 18q21.1 and 18q21.3 respectively (Hahn et al, 1996; Thiagalingam et al, 1996; Takagi et al, 1996; MacGrogan et al, 1997). Colorectal tumours with gains of 20q13 had significantly higher mean S phase fractions than those without, suggesting that amplification of this gene locus may impart a selective growth advantage by increasing the rate of proliferation. Tumours with loss of 4q had lower apoptotic indices than those without, which might indicate that loss of a gene on 4q results in a suppression of apoptosis which again may be advantageous for the overall net growth of a tumour. Other frequent gains seen were of Xq, 8q23–24 and 7p. The *c-myc* (proto)oncogene maps to 8q24 and the *EGFR* gene is located on 7p. Other frequent losses seen were of 1p21–31.1, 5q14–32, 8p2.1-ter, 15qcen-22, and 17p12–13. The *TP53* tumour suppressor gene maps to 17p13.1 and the *APC* tumour suppressor gene to 5q21–22. Loss of heterozygosity (LOH) of the *TP53* gene is known to be implicated in colorectal carcinogenesis, and it has been reported that about 20% of sporadic colorectal carcinomas have LOH in the 5q21–22 region (Solomon et al, 1987). Although we have not examined the present tumour material for LOH at the *APC* locus, it is of interest to note that 27% of the tumours in the present study show deletions of 5q14–32, which covers the *APC* gene locus.

DNA diploid tumours generally had few to no aberrations compared to aneuploid tumours; however, the types of aberrations seen in both groups were similar. Four of 14 DNA diploid tumours in the present study (9%) had no detectable aberrations by CGH, which is in agreement with Ried et al (1996) who reported that 12% of colorectal tumours analysed by CGH had no detectable copy number changes. There are several considerations to take into account in a discussion of DNA diploidy in relation to CGH. The first is that DNA diploid tumours may in fact have no gross chromosomal aberrations, or that the aberrations (gains or losses) are too small to be detected by CGH. Secondly, tumours with only a few aberrations detected by CGH, e.g. gains or losses of one large or two small chromosomes, will be measured as DNA diploid even with high-resolution flow cytometry (Cusick et al, 1990). Finally, it is also possible that gains and losses in DNA diploid tumours balance each other out, so that the net DNA content measured by flow cytometry is ‘normal’, as has been observed in DNA diploid non-Hodgkins lymphomas analysed by CGH (T Stokke, submitted). We are confident that the percentage of contaminating normal cells in DNA diploid tumour samples is not a factor to be taken into consideration when no aberrations were detected by CGH, since we have estimated the percentages of both normal mucosal cells and leucocytes (30–40%) in the respective tumour sections and found them to be similar to those seen in DNA aneuploid tumours. The fact that DNA diploid tumours have so few aberrations compared to aneuploid tumours, even if the actual aberrations are in themselves similar, suggests that genomic instability and possible growth advantages in these tumours result not from acquisition of gross chromosomal aberrations but rather from selection for other (different) types of mutations. This idea is supported by previous studies showing that DNA diploid tumours exhibit microsatellite instability in contrast to DNA aneuploid tumours (Lothe et al, 1993; Remvikos et al, 1995). Roughly 15–20% of sporadic colorectal carcinomas are microsatellite unstable, and half of these are expected to be affected at the *BAX* repeat locus ($(G)_8$ tract of exon 3 of the *BAX* gene) (Rampino et al, 1997). The present tumour series was recently analysed for *BAX* frameshift mutations at this locus (De Angelis et al, 1998), and mutations were detected in three of 42 sporadic tumours analysed; all three tumours were DNA diploid and localized to the right side of the colon. We did not examine the present tumour series for microsatellite instability at other loci. Furthermore, previous studies have suggested that DNA aneuploid and diploid tumours evolve along mostly separate genetic pathways, due to differences between them concerning tumour localization in the colorectum (Delattre et al, 1989; Offerhaus et al, 1992; Lothe et al, 1993), incidence of *TP53* mutations (Kikuchi-Yanoshita et al, 1992; Aaltonen et al, 1993; Meling et al, 1993), and p53 phenotype (Remvikos et al, 1990; Campo et al, 1991; De Angelis et al, 1993). However, the

© Cancer Research Campaign 1999

end result is probably the same – that inactivation of specific tumour suppressor pathways and/or activation of specific oncogenic pathways are selected for despite the utilization of different mechanisms (different aberration pathways) to achieve similar goals. For example, the *BAX* gene, which promotes apoptosis, is mutated in colorectal tumours with microsatellite instability which typically do not have *TP53* mutations (Rampino et al, 1997) and which are DNA diploid (De Angelis et al, 1998). Colorectal tumours with *TP53* mutations and a high number of gross chromosomal aberrations (this work) produce mutant p53 proteins which most likely cannot directly transactivate the *BAX* gene (Miyashita and Reed, 1995). The apoptotic pathway in both types of tumours is thus de-regulated (same selection pressure), but the mechanisms whereby this is effected are different.

DNA hyperdiploid and highly DNA aneuploid tumours do not appear to evolve along separate genetic pathways as was suggested in an earlier study (Meling et al, 1993) since both the type and number of gross chromosomal aberrations per tumour were not significantly different. Finally, the numbers of aberrations in tumours with and without *K-ras* mutations were not significantly different, suggesting that selection for this mutation is not aberration pathway-specific.

The underlying mechanisms responsible for the genomic instability which results in the formation of aneuploid tumours are of interest. De-regulation of $G_2$/M checkpoint networks, cell division/cytokinesis, and apoptotic pathways may lead to the formation and survival of cells with abnormal DNA content, and this may be facilitated by loss of wild-type p53 function via *TP53* mutation in some instances (for review see Shackney and Shankey, 1997). Several models for the generation of DNA aneuploid tumours suggest that they are formed via tetraploidization of diploid cancer cells followed by random chromosome loss (Shackney et al, 1989) or tetraploidization of near-diploid cancer cells (Giaretti, 1993). According to these hypotheses, DNA tetraploid tumours might be expected to have none or few chromosomal aberrations by CGH, since they would have exactly double the diploid or near-diploid chromosome complement. The DNA tetraploid/near-tetraploid tumours in the present study had relatively few aberrations per tumour compared to the aneuploid ones (including both DNA hyperdiploid and highly DNA aneuploid tumours). This is not consistent with tetraploid tumours evolving gradually from diploidy through aneuploidy by sequentially gaining single chromosomes or fragments. Our results suggest that tetraploid tumours evolve by endoreduplication of a diploid or near-diploid tumour cell. These may lose chromosomes to produce DNA aneuploid tumours as is postulated by Shackney et al (1989), but our results cannot elucidate this.

More tumours from males than females had gain of Xq and loss of 18q. Losses of X (four cases) tended to be detected predominantly in tumours from females (three cases). The one tumour from a male with loss of X had a triploid DNA content (DI of about 1.5), which suggests that this tumour simply retained its original copy of X. The results suggest that X may harbour both an oncogene and a tumour suppressor gene(s), and that gene dosage effects of X chromosome genes play a role in colorectal tumorigenesis.

### ACKNOWLEDGEMENTS

We thank Lars Smedshammer for invaluable technical assistance with the preparation of normal metaphase slides. Torstein Scherven and Trond Holmøy are acknowledged for their help with implementation/optimization of the computer hardware and software needed for CGH. Erik Trondsen, MD, and Gunn Iren Meling, MD, PhD, are thanked for providing the colorectal tumours used in this study. We also thank Joe Gray, PhD, Sandy DeVries and Damir Sudar of the Division for Molecular Cytometry Laboratory at the University of California at San Francisco, USA for tips and advice concerning the direct CGH procedure and use of the Scilimage software. This work was supported by the Norwegian Cancer Society.

### REFERENCES

Aaltonen LA, Peltomaki P, Leach FS, Sistonen P, Pylkkanen L, Mechklin J-P, Jarvinen H, Powell SM, Jen J, Hamilton SR, Petersen GM, Kinzler KW, Vogelstein B and de la Chapelle A (1993) Clues to the pathogenesis of familial colorectal cancer. *Science* **260**: 812–816

Andersen SN, Lovig T, Breivik J, Lund E, Gaudernack G, Meling GI and Rognum TO (1997) *K-ras* mutations and prognosis in large-bowel carcinomas. *Scand J Gastroenterol* **32**: 62–69

Arnold N, Hagele L, Walz L, Schempp W, Pfisterer J, Bauknecht T, and Kiechle M (1996) Overrepresentation of 3q and 8q material and loss of 18q material are recurrent findings in advanced human ovarian cancer. *Genes Chromosomes Cancer* **16**: 46–54

Bardi G, Sukhikh T, Pandis N, Fenger C, Kronborg O and Heim S (1995) Karyotypic characterization of colorectal adenocarcinomas. *Genes Chromosomes Cancer* **12**: 97–109

Bauer KD, Bagwell CB, Giaretti W, Melamed M, Zarbo RD, Witzig TE and Rabinovitch P (1993) Consensus review of the clinical utility of DNA flow cytometry in colorectal cancer. *Cytometry* **14**: 486–491

Bigner SH, Bjerkvig R, Læarum OD, Muhlbaier LH and Bigner DD (1987) DNA content and chromosomes in permanent cultured cell lines derived from malignant human gliomas. *Anal Quant Cytol Histol* **9**: 435–444

Bodmer WF, Bailey CJ, Bodmer J, Bussey HJ, Ellis A, Gorman P, Lucibello FC, Murday VA, Rider SH, Scrambler P, Sheer D, Solomon E and Spurr NK (1987) Localization of the gene for familial adenomatous polyposis on chromosome 5. *Nature* **328**: 614–616

Bos JL (1989) Ras oncogenes in human cancer: a review. *Cancer Res* **49**: 4682–4689

Campo E, de la Calle-Martin O, Miquel R, Palacin A, Romero M, Fabregat V, Vives J, Cardesa A and Yague J (1991) Loss of heterozygosity of p53 gene and p53 protein expression in human colorectal carcinomas. *Cancer Res* **51**: 4436–4442

Cher ML, MacGrogan D, Bookstein R, Brown JA, Jenkins RB and Jensen RH (1994) Comparative genomic hybridization, allelic imbalance, and fluorescence in situ hybridization on chromosome 8 in prostate cancer. *Genes Chromosomes Cancer* **11**: 153–162

Cusick, EL, Milton JI and Ewen SWB (1990) The resolution of aneuploid DNA stem lines by flow cytometry: limitations imposed by the coefficient of variation and the percentage of aneuploid nuclei. *Anal Cell Pathol* **2**: 139–148

De Angelis P, Stokke T, Smedshammer L, Lothe RA, Meling GI, Rofstad M, Chen Y and Clausen OPF (1993) p53 expression is associated with a high degree of tumor DNA aneuploidy and incidence of p53 gene mutation, and is localized to the aneuploid component in colorectal carcinomas. *Int J Oncol* **3**: 305–312

De Angelis P, Stokke T, Smedshammer L, Lothe RA, Lehne G, Chen Y and Clausen OPF (1995) P-glycoprotein is not expressed in a majority of colorectal carcinomas and is not regulated by mutant p53 in vivo. *Br J Cancer* **72**: 307–311

De Angelis PM, Stokke T and Clausen OPF (1997) NO38 expression and nucleolar counts are correlated with cellular DNA content but not with proliferation parameters in colorectal carcinomas. *J Clin Pathol Mol Pathol* **50**: 201–208

De Angelis PM, Stokke T, Thorstensen L, Lothe RA and Clausen OPF (1998) Apoptosis and expression of Bax, Bcl-x, and Bcl-2 apoptotic regulatory proteins in colorectal carcinomas, and associations with *p53* genotype/phenotype. *J Clin Pathol Mol Pathol* **51**: 254–261

Delattre O, Law DJ, Remvikos Y, Sastre X, Feinberg AP, Olschwang S, Melot T, Salmon RJ, Validire P and Thomas G (1989) Multiple genetic alterations in distal and proximal colorectal cancer. *Lancet* **2**: 353–356

Eppert K, Scherer SW, Ozcelik H, Pirone R, Hoodless P, Kim H, Tsui LC, Bapat B, Gallinger S, Andrulis IL, Thomsen GH, Wrana JL and Attisano L (1996) MADR2 maps to 18q21 and encodes a TGFβ-regulated MAD-related protein that is functionally mutated in colorectal carcinoma. *Cell* **86**: 543–552

Fearon ER, Hamilton SR and Vogelstein B (1987) Clonal analysis of human colorectal tumors. *Science* **238**: 193–196

 © Cancer Research Campaign 1999

Fearon ER and Vogelstein B (1990) A genetic model for colorectal tumorigenesis. *Cell* **61**: 759–767

Giaretti W (1993) A model on the origin and evolution of DNA aneuploidy. *Int J Oncol* **2**: 165–171

Giaretti W, Monaco R, Pujic N, Rapallo A, Nigro S and Geido E (1996) Intratumor heterogeneity of K-ras2 mutations in colorectal adenocarcinomas. Association with degree of DNA aneuploidy. *Am J Pathol* **149**: 1–9

Hahn SA, Schutte M, Hoque AT, Moskaluk CA, da Costa LT, Rozenblum E, Weinstein CL, Fischer A, Yeo CJ, Hruban RH and Kern SE (1996) DPC4, a candidate tumor suppressor gene at human chromosome 18q21.1. *Science* **271**: 350–353

Heselmeyer K, Macville M, Schrock E, Blegen H, Hellstrom AC, Shah K, Auer G and Ried T (1997) Advanced-stage cervical carcinomas are defined by a recurrent pattern of chromosomal aberrations revealing high genetic instability and a consistent gain of chromosome arm 3q. *Genes Chromosomes Cancer* **19**: 233–240

Hiddemann W, Schumann J, Andreeff M, Barlogie B, Herman CJ, Leif RC, Mayall BH, Murphy RF and Sandberg AA (1984) Convention on nomenclature for DNA cytometry. *Cytometry* **5**: 445–446

Houlston RS and Tomlinson IPM (1997) Genetic prognostic markers in colorectal cancer. *J Clin Pathol Mol Pathol* **50**: 281–288

Ilyas M and Tomlinson IPM (1996) Genetic pathways in colorectal cancer. *Histopathology* **28**: 389–399

Kallioniemi A, Kallioniemi O-P, Sudar D, Rutovitz D, Gray JW, Waldman F and Pinkel D (1992) Comparative genomic hybridization for molecular cytogenetic analysis of solid tumors. *Science* **258**: 818–821

Kallioniemi A, Kallioniemi OP, Citro G, Sauter G, DeVries S, Kerschmann R, Caroll P, and Waldman F (1995) Identification of gains and losses of DNA sequences in primary bladder cancer by comparative genomic hybridization. *Genes Chromosomes Cancer* **12**: 213–219

Kallioniemi OP, Kallioniemi A, Piper J, Isola J, Waldman F, Gray JW and Pinkel D (1994) Optimizing comparative genomic hybridization for analysis of DNA sequence copy number changes in solid tumors. *Genes Chromosomes Cancer* **10**: 231–243

Karhu R, Kahkonen M, Kuukasjarvi T, Pennanen S, Tirkkonen M and Kallioniemi O (1997) Quality control of CGH: impact of metaphase chromosomes and the dynamic range of hybridization. *Cytometry* **28**: 198–205

Kikuchi-Yanoshita R, Konishi M, Ito S, Seki M, Tanaka K, Maeda Y, Iino H, Fukayama M, Koike M, Mori T, Sakuraba H, Fukunari H, Iwama T and Miyaki M (1992) Genetic changes of both p53 alleles associated with the conversion from colorectal adenoma to early carcinoma in familial adenomatous polyposis and non-familial adenomatous polyposis patients. *Cancer Res* **52**: 3965–3971

Korn WM, Oide Weghuis DE, Suijkerbuijk RF, Schmidt U, Otto T, du Manoir S, Geurts van Kessel A, Harstrick A, Seeber S and Becher R (1996) Detection of chromosomal DNA gains and losses in testicular germ cell tumors by comparative genomic hybridization. *Genes Chromosomes Cancer* **17**: 78–87

Lothe RA, Peltomaki P, Meling GI, Aaltonen LA, Nystrom-Lahti M, Pylkkanen L, Heimdal K, Andersen TI, Møller P, Rognum TO, Fosså SD, Haldorsen T, Langmark F, Brøgger A, de la Chapelle A and Børresen A-L (1993) Genomic instability in colorectal cancer: Relationship to clinicopathological variables and family history. *Cancer Res* **53**: 5849–5852

MacGrogan D, Pegram M, Slamon D and Bookstein R (1997) Comparative mutational analysis of DPC4 (Smad4) in prostatic and colorectal carcinomas. *Oncogene* **15**: 1111–1114

Meling GI, Lothe RA, Børresen A-L, Graue C, Hauge S, Clausen OPF and Rognum TO (1993) The *TP53* tumour suppressor gene in colorectal carcinomas. II. Relation to DNA ploidy pattern and clinicopathological variables. *Br J Cancer* **67**: 93–98

Miyaki M, Konishi M, Kikuchi-Yanoshita R, Enomoto M, Igari T, Tanaka K, Muraoka M, Takahashi H, Amada Y, Fukuyama M, Maeda Y, Iwama T, Mishima Y, Mori T and Koike M (1994) Characteristics of somatic mutation of the adenomatous polyposis coli gene in colorectal tumors. *Cancer Res* **54**: 3011–3020

Miyashita T and Reed J (1995) Tumor suppressor p53 is a direct transcriptional activator of the human bax gene. *Cell* **80**: 293–299

Muleris M, Salmon RJ and Dutrillaux B (1990) Cytogenetics of colorectal adenocarcinomas. *Cancer Genet Cytogenet* **46**: 143–156

Nakao K, Shibusawa M, Tsunoda A, Yoshizawa H, Murakami M, Kusano M, Uesugi N and Sasaki K (1998) Genetic changes in primary colorectal cancer by comparative genomic hybridization. *Surg Today* **28**: 567–569

Offerhaus A, Johan G, Defeyter EP, Cornelisse CJ, Tersmette KW, Floyd J, Kern SE, Vogelstein B and Hamilton SR (1992) The relationship of DNA aneuploidy to molecular genetic alterations in colorectal carcinoma. *Gastroenterology* **102**: 1612–1619

Powell SM, Zilz N, Beazer-Barclay Y, Bryan TM, Hamilton SR, Thibodeau SN, Vogelstein B and Kinzler KW (1992) *APC* mutations occur early during colorectal tumorigenesis. *Nature* **359**: 235–237

Rampino N, Yamamoto H, Ionov Y, Li Y, Sawai H, Reed JC and Perucho M (1997) Somatic frameshift mutations in the *BAX* gene in colon cancers of the microsatellite mutator phenotype. *Science* **275**: 967–969

Remvikos Y, Laurent-Puig P, Salmon RJ, Frelat G, Dutrillaux B and Thomas G (1990) Simultaneous monitoring of p53 protein and DNA content of colorectal adenocarcinomas by flow cytometry. *Int J Cancer* **45**: 450–456

Remvikos Y, Vogt N, Muleris M, Salmon RJ, Malfoy B and Dutrillaux B (1995) DNA-repeat instability is associated with colorectal cancer presenting minimal chromosomal rearrangements. *Genes Chromosomes Cancer* **12**: 272–276

Ried T, Knutzen R, Steinbeck R, Blegen H, Schrock E, Heselmeyer K, du Manoir S and Auer G (1996) Comparative genomic hybridization reveals a specific pattern of chromosomal gains and losses during the genesis of colorectal tumors. *Genes Chromosomes Cancer* **15**: 234–245

Rognum TO, Lund E, Meling GI and Langmark F (1991) Near diploid large bowel carcinomas have better five-year survival than aneuploid ones. *Cancer* **68**: 1077–1081

Shackney SE and Shankey TV (1997) Common patterns of genetic evolution in human solid tumors. *Cytometry* **29**: 1–27

Shackney SE, Smith CA, Miller BW, Burholt DR, Murtha K, Giles HR, Ketterer DM, and Pollice AA (1989) Model for the genetic evolution of human solid tumors. *Cancer Res* **49**: 3344–3354

Solomon E, Voss R, Hall V, Bodmer WF, Jass JR, Jeffreys AJ, Lucibello FC, Patel I and Rider SH (1987) Chromosome 5 allele loss in human colorectal carcinomas. *Nature* **328**: 616–619

Takagi Y, Kohmura H, Futamura M, Kida H, Tanemura H, Shimokawa K and Saji S (1996) Somatic alterations of the DPC4 gene in human colorectal cancers in vivo. *Gastroenterology* **111**: 1369–1372

Tanner MM, Tirkkonen M, Kallioniemi A, Collins C, Stokke T, Karhu R, Kowbel D, Shadravan F, Hintz M, Kuo, W-L, Waldman FM, Isola JJ, Gray JW and Kallioniemi O-P (1994) Increased copy number at 20q13 in breast cancer: defining the critical region and exclusion of candidate genes. *Cancer Res* **54**: 4257–4260

Thiagalingam S, Lengauer C, Leach FS, Schutte M, Hahn SA, Overhauser J, Willson JK, Markowitz S, Hamilton SR, Kern SE, Kinzler KW and Vogelstein B (1996) Evaluation of candidate tumour suppressor genes on chromosome 18 in colorectal cancers. *Nat Genet* **13**: 343–346

Tirkkonen M, Tanner M, Karhu R, Kallioniemi A, Isola J and Kallioniemi O-P (1998) Molecular cytogenetics of primary breast cancer by CGH. *Genes Chromosomes Cancer* **21**: 177–184

Tribukait B, Granberg-Ohman I and Wijkstrom H (1986) Flow cytometric DNA and cytogenetic studies in human tumors: a comparison and discussion of the differences in modal values obtained by the two methods. *Cytometry* **7**: 194–199

Vindeløv LL, Christensen IJ and Nissen NI (1983) A detergent-trypsin method for the preparation of nuclei for flow cytometric DNA analysis. *Cytometry* **3**: 323–327

Vogelstein B, Fearon ER, Hamilton SR, Kern SE, Preisinger AC, Leppert M, Nakamura Y, White R, Smits AMM and Bos JL (1988) Genetic alterations during colorectal tumor development. *N Engl J Med* **319**: 525–532

© Cancer Research Campaign 1999
